# Cycle thresholds and discordant 2-step testing for Clostridioides Difficile: Comparison of clinical characteristics and outcomes among discordant and true positive results

**DOI:** 10.1017/ash.2026.10375

**Published:** 2026-05-11

**Authors:** Michael Rossi, Emerald O’Rourke, Tao Hong, Sara F. Geffert, Tiffany L. Chargualaf, Andrea Collins, Joshua Ray Tanzer, Francine Touzard-Romo

**Affiliations:** 1 Warren Alpert Medical School of Brown University, USA; 2 Brown University Health, USA; 3 Newport Hospital, USA; 4 Rhode Island Hospital, USA

## Abstract

**Objective:**

To determine if Toxin B polymerase chain reaction (PCR) cycle threshold (Ct) can help identify which patients with discordant clostridioides difficile (CD) PCR and enzyme immunoassay testing (EIA) may benefit from treatment.

**Design:**

Retrospective case series.

**Setting:**

Multi-hospital tertiary Academic system.

**Patients:**

All admitted adult patients with positive CD PCR over a 6 month period (n = 214). Patients with positive Stool PCR for other pathogens were excluded.

**Methods:**

Chart review of all eligible patients. CD testing results, patient demographics, underlying comorbidities, symptoms prompting testing, risk factors for CD, markers of severe disease, treatment regimens, and clinical outcomes including: 30-day all-cause mortality and re-admission, and 60-day CDI repeat testing and treatment were collected.

**Results:**

Significant association between mortality and lower Ct values among discordant patients who were not treated compared to those who were (P = 0.042). The association between Ct values and 30-day readmission was significantly stronger among untreated patients compared to those who were treated (P = .034). A similar trend was observed among non-severe, untreated patients with low Ct values and subsequent CDI treatment within 60 days (CT < 26, 49% vs. CT > 26, 18%, P = .063).

**Conclusions:**

Untreated patients with lower cycle thresholds had worse clinical outcomes compared to untreated patients with higher Ct, regardless of disease severity. A lower PCR Ct can support treatment in patients with clinical disease, positive PCR and negative toxin EIA.

## Background

The diagnosis of Clostridioides difficile infection (CDI) is challenging. Despite guideline-directed multistep testing algorithms and diagnostic stewardship, there is no definite method that differentiates colonization vs true infection (Mcdonald, Polange).

In our health care system, a real-time polymerase chain reaction test (PCR) for Toxin B gene, paired with an electronic medical record nudge for appropriate testing, reflexes to a Toxin A/B enzyme immunoassay (EIA) if positive. PCR is a highly sensitive test for presence of Clostridioides difficile (CD), but does not test for active toxin production, whereas the EIA has lower sensitivity and higher specificity. Given these testing characteristics, concordant results (PCR+/EIA+) are considered true infection, while discordant results (PCR+/EIA−) can represent colonization, true infection in patients with low level of toxin production, or false negative EIA results. However, most patients with discordant results in our system, and globally, are treated (Lenggenhager). Accurately identifying which patients with discordant testing need treatment is crucial to avoid overtreatment, unnecessary costs, selection for Vancomycin Resistant Enterococci, and anchoring on incorrect diagnoses. Furthermore, the National Healthcare Safety Network may update their surveillance definition of CDI to account for treatment which will affect Centers for Medicare and Medicaid (CMS) quality ranking and hospital reimbursement. Quantitative PCR Cycle threshold (Ct) inversely correlates with the amount of target DNA present and in CDI has been shown to be predictive of free toxin detection (Kamboj, Mah) and thus has been posited as a tool in treatment decisions.

Our study compares clinical characteristics, Ct values, and outcomes among patients treated and not treated for CDI to assess how Ct interacts with clinical outcomes amongst patients with discordant and concordant testing.

## Methods

After Institutional Review Board approval, we performed a retrospective chart review of all adult patients (≥18-year-old) who had a positive CD PCR result and were admitted to our 3-hospital health system between June 01 and October 31, 2023. CD PCR and Ct results were obtained by *Cepheid GeneXpert,* and Toxin A and B EIA results were obtained by *Meridian Bioscience Immunocard*. We collected data on patient demographics, underlying comorbidities, symptoms prompting testing, risk factors for CDI, markers of severity, treatment, and clinical outcomes including: 30-day all-cause mortality and re-admission, and 60-day CDI repeat testing and treatment. Infectious Diseases Society of America-Society for Healthcare Epidemiology of America definitions were utilized, whereby severe disease is defined as creatinine >1.5 mg/dL or white blood cell count >15,000/L and fulminant is severe disease with either hypotension or megacolon. Treatment was defined as completing at least 5 days of oral vancomycin, metronidazole or fidaxomicin. Patients with positive Stool PCR for a non-CD pathogen were excluded.

### Statistical methods

We focused on comparing the associations between Ct value and health outcomes between patients who were treated for CDI and patients who were not treated for CDI. Generalized linear mixed effects modeling was used to estimate the likelihoods of each health outcome based on whether the patient was treated and Ct value. Each outcome was categorical, so a binomial distribution with logit link was used. The focus of interpretation was on the interaction term between Ct value and patient population. The comparison emphasized patients who had discordant test results (ie, PCR+/EIA−) who were and were not treated.

Aware that clinical judgment may have been relevant to provider decision making on who should be treated when results were discordant, the analysis also included an interaction term with disease severity (severe/fulminant or not severe). This addresses the concern that a severe patient with discordant results may be more likely to be treated regardless of whether they have true CDI. Lastly, a random effect was used.

As contextual information, we also reviewed descriptive statistics on groups based on treatment status and test results (concordant or discordant). Specifically, we examined averages or percentages on relevant clinical characteristics, as well as 95% confidence intervals, to compare patient groups. To simplify interpretation, we calculated *P*-values testing the assumption of no difference in group averages for group comparisons we found insightful. This does not amount to a test of our research aims, but did help to understand the ways in which patients differed from one another.

## Results

A total of 214 patients were identified who met inclusion criteria. 62 had positive toxin EIA (concordant) and 152 had negative toxin EIA (discordant). All concordant patients were treated and of the discordant patients, 113/148 (76%) were treated. Demographic characteristics were similar among groups (Table 1). Abdominal CT scan was performed in 135 patients and concordant patients had abnormal findings significantly more often than discordant patients (*P* = .032). In clinical presentation, amongst discordant patients treated versus not treated, there was no significant difference in frequency of 3 or more bowel movements in the last 24 hours (*P* = .213), nor abnormal CT scan (*P* = .201). There was also no difference between these groups in their likelihood of receiving PPI nor antibiotics in the preceding 14 days (*P* = .666 and .107 respectively). Lastly, severe infection was found in 21% of not-treated discordant patients and 39% of treated discordant patients, a significant difference (*P* = .024). Mean Cycle threshold for concordant patients was significantly lower (24.15) compared to the discordant patients irrespective of severity; for both treated (28.76) and not treated (29.01) (*P* < .001).

**Table 1.** Demographics and clinical characteristics among PCR+/EIA and PCR/EIA−by treatment

Trait	PCR+/EIA−, not treated	PCR+/EIA−, treated	PCR+/EIA+, treated
Total number of patients	39	113	62
**Demographics**			
Age (years)	61 [58, 66]	65 [63, 68]	70 [67, 74]
Female gender (%)	46 [31, 62]	53 [43, 62]	53 [42, 65]
**Clinical presentation**			
Three or more bowel movements in 24 hours (%)	62 [43, 80]	73 [62, 82]	79 [67, 90]
Fever (%)	23 [10, 38]	14 [8, 21]	19 [10, 27]
Hypotension (%)	18 [8, 33]	20 [14, 28]	21 [12, 31]
Abdominal CT performed (%)	56 [41, 72]	66 [58, 73]	61 [50, 73]
Abnormal CT findings (%)	29 [10, 48]	40 [31, 53]	67 [53, 81]
Vasopressors required (%)	18 [8, 33]	5 [3, 11]	6 [2, 12]
**Medical history**			
Laxative use within 48 hours of testing (%)	10 [3, 21]	16 [10, 22]	13 [5, 23]
Antibiotics within 14 days of testing (%)	51 [33, 67]	67 [57, 76]	84 [72, 93]
PPI Use within 14 days of testing (%)	50 [33, 65]	46 [37, 56]	69 [57, 79]
History of PCR + test (%)	42 [26, 54]	25 [18, 33]	27 [18, 38]
History of colon surgery (%)	3 [0, 8]	3 [0, 6]	5 [0, 11]
**Comorbidities**			
Alcohol use disorder (%)	29 [15, 44]	21 [13, 29]	16 [8, 26]
End-stage renal disease (%)	10 [3, 21]	7 [3, 12]	8 [2, 15]
Cirrhosis (%)	15 [5, 28]	10 [5, 15]	8 [2, 16]
Inflammatory bowel disease (%)	8 [0, 16]	6 [2, 12]	6 [2, 13]
Immunosuppression (%)	23 [13, 38]	20 [13, 28]	20 [10, 30]
**Lab testing**			
Stool panel performed (%)	54 [37, 69]	55 [43, 68]	47 [34, 58]
White blood cell count (M)	12.04 [9.52, 14.8]	8.54 [7.64, 9.49]	14.35 [12.17, 16.54]
Albumin (M)	2.98 [2.76, 3.18]	3.49 [3.34, 3.67]	3.02 [2.83, 3.19]
Serum creatinine (M)	1.43 [0.95, 2.4]	1.35 [1.07, 1.76]	1.52 [1.2, 1.93]
**C diff treatment**			
Fulminant infection (%)	18 [8, 33]	6 [3, 12]	6 [2, 12]
Severe infection (%)	21 [10, 34]	39 [31, 48]	55 [42, 66]
Cycle threshold (M)	29.01 [27.48, 30.5]	28.76 [27.89, 29.68]	24.15 [23.41, 24.85]
Cycle threshold <26 (%)	31 [18, 46]	37 [27, 45]	79 [71, 89]

**Figure 1.**
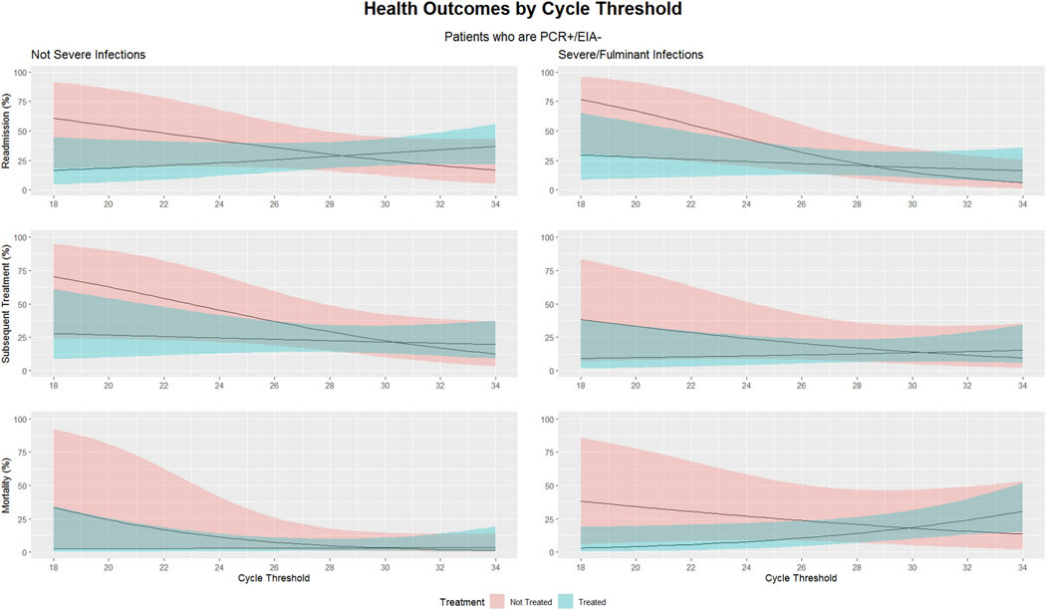
Plotted are the model implied percentages of each outcome by Ct for discordant patients: comparing patients who were and were not treated. The shading represents 95% confidence interval.

Overall, 27% of patients were readmitted within 30 days and 18% were treated within 60 days from the index diagnosis. Untreated patients with lower Ct trended toward worse mortality outcomes overall (28%) [9%–62%] vs 16% [4%–48%] (*P* = .442). In Figure 1, statistical analysis focused on the difference in magnitudes of association between treated and untreated patients. Untreated, discordant patients, with severe disease and lower Ct values were significantly more likely to be readmitted within 30 days compared to higher Ct values (CT < 26, 48% vs. CT > 26, 11%, OR = 0.14, *P* = .014). Relatedly, untreated, discordant patients with non-severe disease and Ct <26 trended towards higher readmission rates compared to those with Ct >26 (44% [20%–72%] vs 22% [9%–43%], OR = 0.35, *P* = .154) Overall, the association between Ct values and 30-day readmission was significantly stronger among untreated patients compared to those who were treated (*P* = .034). A similar trend was observed among non-severe, untreated patients with low Ct values and subsequent CDI treatment within 60 days (CT < 26, 49% vs. CT > 26, 18%, *P* = .063).

Mortality overall was low. In non-severe, discordant patients that were treated, mortality was the same for both Ct above and below 26 (3%) [0%–16%] vs 3% [1%–13%]. In those same patients who were not treated, mortality was 13% [2%–50%] vs 2% [0%–14%], (OR = 0.15, *P* = .136) when comparing Ct < 26 and >26 respectively. Across all discordant patients there was a significantly stronger association between mortality and lower CT values among discordant patients who were not treated compared to those who were treated (*P* = .042).

## Discussion

As 2-step CDI testing algorithms become standard and CMS reporting definitions change, determining which patients warrant treatment is crucial. Among PCR+/EIA−patients, there is no reliable test to differentiate CDI from colonization. We posited that PCR Ct values might help support providers in treatment decisions.

Several studies have shown lower Ct values for CD PCR inversely correlate with disease severity and clinical outcomes, though there is conflicting data on test performance, optimal Ct value, and the ability to use Ct in treatment decisions remains unclear (Reigadas, Choi, Bonacorsi, Crone, Kamboj, Mah). Senchyna et al found an optimal Ct of 26.35 to predict toxin production and Kamboj et al found a Ct of 28 to best predict severe CDI, while Garvey et al found that a Ct ≤ 26 was associated with higher 30-day mortality and severe CDI. In the Hogan et al quasi-experimental study, changing result reporting to positive PCR/negative toxin using Ct > 27.5 found that only 15% of Ct negative patients were treated, and 30-day mortality and 7-day diarrhea resolution was non-inferior when compared to the Ct-toxin positive patients. However, subsequent PCR+/Ct-toxin positive conversion and hospital length of stay did not meet non-inferiority threshold.

We found concordant patients had lower Ct irrespective of severity compared to discordant patients and an overall linear correlation between worse outcomes and lower cycle threshold. This was seen particularly among untreated discordant patients with lower Ct.

Regarding 30-day readmission, consistent with our hypothesis, in discordant patients the negative correlation between Ct values and 30-day readmission was significantly stronger among untreated patients compared to those who were treated. Thus, lower Ct patients were more likely to be re-admitted, but treating this population decreased the risk of re-admission. The mortality outcomes in discordant patients with lower Ct were also better when treated. The reduced association between lower Ct values and worse outcomes among treated patients suggests a potential benefit of treating discordant patients with lower Ct values.

In contrast, untreated discordant patients with higher Ct were not more likely to have worse outcomes than those who were treated, which can support not treating this group of patients. These correlations held true for both severe and non-severe disease. This holding true for severe disease may be due to patients meeting criteria for severe disease due to other non-CDI conditions.

Based on prior literature, we had posited using a practical Ct cutoff of 26 to aid in the decision of which discordant patients to treat. Numerically, in this retrospective analysis, the actual optimal cutoff Ct varied from 28 to 30 depending on outcome, whereby above these Ct values, treatment did not affect outcomes. Figure 1 is a graphical representation of this and visually represents the overall trends of worse outcomes in untreated discordant patients with low cycle thresholds as compared to those who were treated. Given the continuous nature of Ct as a variable and the risk-benefit calculation of over versus under treatment in an individual patient, the optimal clinically useful cutoff is not clear. Overall, in conjunction with prior literature, this data suggests that as Ct moves away from 26 to 30 and toward the extremes, it can be used with increasing confidence in treatment decisions. Ct values in the range of 26–30 may be viewed as more indeterminate, and rather than focusing on an absolute number cutoff, we would suggest Ct be a pragmatic adjunctive tool and data point to aid in treatment decisions in individual discordant patients.

Further complicating Ct utilization is concern that multiple variables affect Ct such as volume of sample collection, time since infection onset, and inter-laboratory reliability which are difficult to standardize (Hilt). Pollock et al. found that toxin concentration, which Ct can be a proxy for, did not seem to help distinguish true infections from colonization. Given the retrospective nature of this study, we could not control for treatment decisions of each individual provider and there might be unique factors that were not captured in our review. Furthermore, given that most discordant patients were treated and mortality was low, our sample size of non-treated discordant patients is smaller and potentially underpowered. Also, special populations such as immunosuppressed or inflammatory bowel disease patients were not investigated separately and may have different relationships between Ct, outcomes, and severity. In the discordant untreated population, there is a percentage of patients with hypotension, fever and pressor requirement which may have been caused by non-CDI processes which contributed to the decision not to treat and affected overall clinical outcomes. Given that definitions for severe and fulminant CDI were based on laboratory and clinical markers, alternative clinical diagnoses may not have been captured. Prospective studies would be beneficial in further characterizing the optimal implementation of Ct ranges in treatment algorithms.

Overall, lower Ct correlated with true disease and worse clinical outcomes among EIA negative patients, regardless of disease severity. Discordant patients with higher Ct who were not treated did not have worse outcomes compared to those who were treated and discordant patients with lower Ct had better outcomes when treated. Given the limitations of Ct and ongoing research into optimal use of Ct and standardization, Ct in CDI will not replace clinical evaluation and judgment, but can serve as an adjunctive tool and additional data point to interpret in making treatment decisions in discordant patients.
